# Neurocognition Function of Patients With Bipolar Depression, Unipolar Depression, and Depression With Bipolarity

**DOI:** 10.3389/fpsyt.2021.696903

**Published:** 2021-07-28

**Authors:** Zhe Lu, Yingtan Wang, Guanglei Xun

**Affiliations:** ^1^Cheeloo College of Medicine, Shandong University, Jinan, China; ^2^Peking University Sixth Hospital, Institute of Mental Health, Peking University, Beijing, China; ^3^Department of Mental Health, Jining Medical University, Jining, China; ^4^Shandong Mental Health Center, Jinan, China

**Keywords:** unipolar depression, bipolar depression, bipolarity, THINC-it, Wisconsin Card Sorting Test, continuous performance test

## Abstract

Much evidence shows that some *Diagnostic and Statistical Manual of Mental Disorders*, fifth edition (DSM-5)-defined unipolar depression (UD) with bipolarity manifests bipolar diathesis. Little is known about the cognitive profiles of patients with depression with bipolarity (DWB). The study aimed to investigate the differences in cognitive profiles among patients with bipolar depression (BD), major depressive disorder (namely, UD), and DWB. Drug-naïve patients with BD, UD, and DWB and healthy controls (HC) were recruited (30 cases in each group). Cognitive function was evaluated by THINC-it (THINC-intelligent tool), Wisconsin Card Sorting Test (WCST), and continuous performance test (CPT). For THINC-it, no significant differences of the Z-scores in both objective and subjective factors were found between the DWB group and BD group, but the Z-scores in the BD group were significantly lower than those in the UD group. For WCST, significant differences were found between the BD group and DWB group in the number of responses, categories completed, trails to completed first category, perseverative responses, and perseverative errors. All the indices of WCST in the DWB group were significantly worse than those in the UD group except for trails to completed first category and total number of response correct. For CPT, only scores of leakage responses and false responses in the four-digit number in the BD group and DWB group were significantly higher than those in the UD group; no significant difference was found between the BD group and DWB group. The results indicated that patients with DWB might perform differently from those with UD but similarly to those with BD with cognition impairment.

## Introduction

Bipolar disorder is a severe mental illness with high morbidity, high recurrence rate, and high disability, which brings the fearful burden of disease to patients, their families, and society (1). The WHO World Mental Health Survey Initiative about bipolar spectrum disorder (BSP) showed that the aggregate lifetime prevalence of BSP was 2.4% (0.6% for bipolar I disorder, 0.4% for bipolar II disorder, and 1.4% for subthreshold bipolar disorder) (2).

Compared with the *Diagnostic and Statistical Manual of Mental Disorders*, fourth edition (DSM-IV), DSM-5 expanded the connotation of bipolar disorder and divided mood disorders into bipolar disorder and depressive disorder in consideration of the differences of symptoms, genetic features, and clinical characteristics between the two disorders, which improved the diagnostic accuracy (3). Although, many differences between the two disorders have been detected like age of onset, the diagnostic rate of bipolar disorder is still lower than expected, especially for bipolar disorder type II. A previous study showed that 69% BD patients were misdiagnosed within 1 year of the onset of symptoms, with the most frequent misdiagnosis being unipolar disorder (UD) (4). Okasha et al. (5) used the Hypomania Checklist-32 to estimate the frequency of bipolar disorder among patients with a major depressive episode (MDE), and the result showed that 62% of patients diagnosed with unipolar depression were positive on the bipolar screening.

Moreover, growing evidence suggests that the DSM criteria for bipolar II disorder are so strict that some individuals who express varying manifestations of bipolar syndrome to a lesser extent are excluded (6–8). However, identifying these subthreshold individuals is of clinical importance because they are more likely to commit suicide (9), suffer more recurrent depressive episodes (10), and convert into bipolar disorders than individuals with UD (11).

Hence, the concept of bipolarity is of great importance and relevance to clinicians to promote judicious diagnosis and the use of antidepressants. Akiskal et al. (12–15) previously proposed a construct of soft bipolar spectrum (SBP) beyond bipolar I and bipolar II disorder, including bipolar II1/2 (depression with the cyclothymic temperament), bipolar III (depression with hypo/mania associated with antidepressants), and bipolar IV (depression with the hyperthymic temperament), which improved the validity of current diagnosis and was validated in the French National epidemiology of depression study. Furthermore, Ghaemi et al. (16) redefined the BSP according to some indicators of bipolarity. Therefore, although, we cannot give a bipolar disorder diagnosis to the patients with the above symptoms based on DSM-5, clinicians should also keep these “bipolarity pointers” in mind and prescribe antidepressants more charily.

It is noteworthy that cognitive impairment is a core feature of BD (17) and UD (18). Previous studies indicated that cognitive function decreased significantly in BD and UD patients during acute episodes and might persist into euthymic periods (19–21). Recently, a study conducted by Lin et al. about the differences in neurocognitive function among bipolar I disorder, bipolar II disorder, and SBP disorder showed that patients with SBP differ from patients with strict UD. Moreover, the study also showed that cognitive deficits of BSP proposed by Ghaemi were similar to the SBP (22). However, little is known about cognitive deficits in individuals with depression with bipolarity (DWB) and the extent to which they perform differently from those with UD and BD. Therefore, we hypothesized that individuals with DWB might perform differently from those with UD but similarly to those with BD. The goal of the present study was to explore neurocognitive characteristics of DWB patients.

## Materials and Methods

### Subjects

Patients with MDE were recruited in Shandong Mental Health Center, from May 2019 to January 2020. Inclusion criteria and exclusion criteria were as follows.

*Inclusion criteria for patients*: (1) DSM-5-diagnosed MDE; (2) not treated with psychotropic or any other somatic therapies and psychotherapy within 2 months; (3) aged 18–45 years, Han Chinese; (4) education level of junior high school or above; (5) scores of Hamilton Rating Scale for Depression-17 (HAMD-17) ≥17, scores of Young Mania Rating Scale (YMRS) <6; and (6) understanding research content and providing written informed consent.

*Inclusion criteria for healthy controls* (HC): (1) without any mental disorders and family history of mental disorders; (2) aged 18–45 years, Han Chinese; (3) education level of junior high school or above; (4) HAMD-17 <7 and YMRS <6; and (5) understanding research content and providing written informed consent.

*Exclusion criteria applied to all participants*: (1) with other mental disorders; (2) history of organic brain diseases or brain trauma; (3) severe physical disease that might interfere with the study evaluations; (4) color blindness or color weakness; (5) pregnancy or lactation; and (6) alcohol or other substance usages.

After obtaining written consent, two senior psychiatrists (who had been in practice for more than 10 years) conducted clinical interviews independently applying the Structured Clinical Interview for DSM-IV-TR Axis I Disorders Patient Edition (Chinese version) to confirm the diagnoses. The inter-rater reliability between the two interviewers was high (kappa value > 0.9).

Patients diagnosed with bipolar I or II disorder based on DSM-5 were in the BD group. Patients diagnosed with major depressive disorder (MDD) based on DSM-5 received another interview by a senior postgraduate to detect bipolarity. Those who met the criteria of bipolarity (as follows) were categorized into the DWB group, and others were in the UD group.

*Criteria of bipolarity* (16): (i) at least one MDE; (ii) no spontaneous hypomanic or manic episode; (iii) a family history of bipolar disorder in the first-degree relative, (iv) plus at least two items from criterion; (iv) if no family history of bipolar disorder is present, six of the following nine criteria are needed: (1) hyperthymic personality (at baseline, no depressed state); (2) recurrent MDEs (>3); (3) brief MDEs (on average, <3 months); (4) atypical depressive symptoms (DSM-5); (5) psychotic MDEs; (6) early age of onset of MDE (<age 25); (7) postpartum depression; (8) antidepressant “wear-off” (acute but not prophylactic response); and (9) lack of response to ≥3 antidepressant treatment trials. The present study adopted Ghaemi's criterion of bipolarity except for a mild modification in criterion (iii). Because antidepressant-induced mania or hypomania has been sufficient to establish a bipolar diagnosis according to DSM-5, it was deleted from criterion (iii) in Ghaemi's criterion.

The study protocol was approved by the Clinical Research Ethics Committee of Shandong Mental Health Center and is compliant with the Code of Ethics of the World Medical Association (Declaration of Helsinki). Informed written consent was obtained from all participants or their legal guardians after a complete and extensive description.

### Evaluation Instruments and Assessment

The severity of symptoms was assessed with HAMD-17, Hamilton Rating Scale for Anxiety (HAMA), YMRS, and Clinical Global Impression Scale-Severity (CGI-S). The Mood Disorder Questionnaire (MDQ) was completed by participants.

Neurocognitive function was assessed with THINC-intelligent tool (THINC-it), Wisconsin Card Sorting Test (WCST), and continuous performance test (CPT). THINC-it is a recently validated, computerized cognitive assessment tool (http://thinc.progress.im/en) containing variants of commonly used and well-established measures of cognition. It can assess the objective [digit symbol substitution test (DSST); choice reaction time task (CRT); trail-making test B, (TMT-B); and N-back memory task (N-Back)] and subjective cognitive function (Perceived Deficits Questionnaire 5) simultaneously and can be self-administered by the patient (23). Standardized Z-scores were calculated to compare performance on both objective and subjective cognitive assessments on the THINC-it (24). WCST was used to assess executive function; the indices from WCST include total number of response (TR), number of categories completed (CC), total number of response correct (RC), total number of response errors (RE), trails to completed first category (TCFC), perseverative responses (PR), perseverative errors (PE), non-perseverative errors (nPE), and percent conceptual level responses (PCLR). CPT was applied to assess sustained attention; the indices from CPT include leakage responses (LR), false responses (FR), and mean reaction time (MRT) of three levels (two-digit, three-digit, and four-digit numbers).

### Statistical Analysis

All data were analyzed with SPSS Statistics, Version 26 (Chicago, IL, USA). The Kolmogorov–Smirnov test was used to test the normal distribution of the measurement data. One-way analysis of variance (ANOVA) was used to compare the differences among groups for normal distribution data, and the Kruskal–Wallis test was performed for non-normal distribution data. Chi-square test or Fisher's exact test was conducted to analyze categorical variables. Group differences in THINC-it, WCST, and CPT indices were tested by analysis of covariance, with age, sex, and education years as covariates. The Bonferroni test as the *post-hoc* multiple comparison was used to identify the differences among four groups. In particular, the *post-hoc* comparison among three patient groups were adjusted by age, sex, education years, age of onset, number of episodes, course of disorder, duration of current depressive episode, and HAMA and HAMD scores. A two-tailed *p*-value of <0.05 was considered statistically significant.

## Results

### Demographic and Clinical Characteristics

Thirty participants in each group (BD, UD, DWB, and HC) were enrolled in this study. There was no significant difference in sex, age, education years, and body mass index (BMI) among the four groups. The differences in age of onset among three patient groups were significant; however, pairwise comparison showed no significant difference after the Bonferroni adjustment. There was no significant difference in the number of episodes between the UD group and DWB group, while the number of episodes in the BD group was significantly higher than that in the UD group and DWB group. Course of disorder in the BD group was significantly higher than that in the UD group, while significant differences in the course of disease were found neither between the DWB group and UD group nor between the BD group and DWB group. No significant difference was found in the duration of the current depressive episode among three patient groups. There was no significant difference in MDQ scores between the BD and DWB groups, while the MDQ in the BD group and DWB group was significantly higher than that of the UD group. There was no significant difference in family history, course of disorder, whether with psychotic symptoms, CGI-S, scores of HAMD-17, and HAMA among the three patient groups (Table 1).

**Table 1:** Demographic and clinical characteristics of participants.

	**BD (***n*** = 30)**	**UD (***n*** = 30)**	**DWB (***n*** = 30)**	**HC (***n*** = 30)**	***Z/χ*** ^**2**^	***p***
	**Median (IQR 25–75)/mean ± SD**	**Median (IQR 25–75)/mean ± SD**	**Median (IQR 25–75)/mean ± SD**	**Median (IQR 25–75)/mean ± SD**		
Sex (male/female)	16/14	13/17	14/16	15/15	0.667	0.881
Age (years)	28.5 (17, 33)	21 (19, 40)	21 (17, 31)	24.5 (19, 29.5)	2.826	0.419
Education years (years)	13.5 (11, 16)	12 (9, 15)	13 (11, 15.25)	13 (12, 15.25)	2.448	0.485
BMI	23.775 (22.4, 25.47)	21.975 (19, 26.93)	23.87 (20.1575, 25.08)	20.705 (23.145, 24.8275)	1.177	0.785
Family history (positive/negative)	4/26	4/26	5/25	NA	0.180	0.914
Age of onset (year)	17.5 (15.75, 27)	20 (18, 34)	17 (16, 22.75)	NA	7.924	0.019
Number of episodes	2.5 (2, 3)	1 (1, 2)	1 (1, 2)	NA	30.212	<0.001
Course of disorder (month)	21 (12, 36)	11 (4, 16)	14 (1, 51)	NA	11.572	0.003
Duration of current episode (month)	2.5 (1, 6)	6 (1.75, 13)	5.5 (1, 21)	NA	3.255	0.196
Whether with psychotic symptom (yes/no)	5/25	2/28	6/24	NA	2.338	0.311
CGI-S	5 (4, 6)	5 (4.75, 6)	5 (4, 6)	NA	0.396	0.820
HAMD-17	23.83 ± 2.96	24.13 ± 4.55	24.20 ± 6.01	NA	0.052	0.949
HAMA	27.50 ± 3.36	24.33 ± 5.01	25.20 ± 8.05	NA	2.380	0.099
MDQ	6.17 ± 2.52	2.73 ± 1.62	5.50 ± 2.75	NA	48.149	<0.001

*BD, bipolar depression; UD, unipolar depression; DWB, depression with bipolarity; HC, healthy control; BMI, body mass index; GCI-S, Clinical Global Impression Scale-Severity; HAMD-17, Hamilton Rating Scale for Depression-17; HAMA, Hamilton Rating Scale for Anxiety; MDQ, Mood Disorder Questionnaire; SD, standard deviation; IQR, interquartile range*.

### THINC-Intelligent Tool

#### Objective Cognition

The differences of Z-scores between DWB and BD, UD and DWB, and UD and HC were not significant, while Z-scores in the BD group (*p* = 0.027) were significantly lower than those in the UD group, and Z-scores in the BD group (*p* < 0.001) and DWB group (*p* < 0.001) were lower than those in the HC. As to each item of objective component, the Z-scores of all items in the three patient groups were significantly lower than those in the HC group except for the CRT. There was no significant difference in Z-score of N-Back among the three patient groups. The Z-scores of DSST and TMT-B in the BD group were significantly lower than those in the UD group and DWB group (Table 2).

**Table 2:** Comparison of THINC-it among patients with bipolar depression, unipolar depression, depression with bipolarity, and healthy controls (mean ± SD).

	**BD (***n*** = 30)**	**UD (***n*** = 30)**	**DWB (***n*** = 30)**	**HC (***n*** = 30)**	***F*** **^1^**	***p*** **^1^**	***post-hoc*** **^1^**	***F*** **^2^**	***p*** **^2^**	***post-hoc*** **^2^**
Objective part	−2.85 ± 2.98	−1.40 ± 1.71	−2.51 ± 2.04	0.00 ± 1.00	11.570	<0.001	BD < UD; BD, DWB < HC	5.290	0.007	BD < UD
DSST	−3.97 ± 1.62	−2.36 ± 2.31	−2.80 ± 0.71	0.00 ± 1.00	35.371	<0.001	BD < UD, DWB < HC	6.095	0.003	BD < DWB, UD
CRT	−0.37 ± 2.00	−0.72 ± 2.18	−0.41 ± 1.57	0.00 ± 1.00	0.905	0.441		0.630	0.535	
TMT	−2.71 ± 1.90	−1.64 ± 1.98	−1.36 ± 0.86	0.00 ± 1.00	15.765	<0.001	BD < DWB; BD, UD, DWB < HC	8.594	<0.001	BD < DWB
N-Back	−2.21 ± 1.74	−1.46 ± 1.85	−1.40 ± 1.84	0.00 ± 1.00	9.252	<0.001	BD, UD, DWB < HC	1.084	0.343	
Subjective part	−4.96 ± 2.05	−3.09 ± 2.32	−4.46 ± 2.64	0.00 ± 1.00	33.208	<0.001	BD < UD; BD, UD, DWB < HC	5.611	0.005	BD, DWB < UD

*THINC-it, THINC-intelligent tool; DSST, digit symbol substitution test; CRT, choice reaction time task; TMT, Trail making test B; N-Back, N-back memory task; BD, bipolar depression; UD, unipolar depression; DWB, depression with bipolarity; HC, healthy control*.

1 *Age, sex, education years as covariates among four groups*.

2 *Age, sex, education years, age of onset, number of episodes, course of disorder, duration of current depressive episode, and HAMA and HAMD-17 scores as covariates among three patient groups*.

#### Subjective Cognition

The difference of Z-score between the DWB group and BD group, and the DWB group and UD group was not significant, while Z-scores in the BD group (*p* = 0.008) were significantly lower than those in the UD group; and Z-scores in the three patient groups were significantly lower than those in the HC. The Z-scores in the DWB group were significantly lower than those in the UD (*p* = 0.029) after being adjusted by age, sex, education years, age of onset, number of episodes, course of disorder, duration of current depressive episode, and HAMA and HAMD-17 scores (Table 2).

### Wisconsin Card Sorting Test

There was no significant difference in TR, PR, and PE between the BD and DWB groups. TR (BD, *p* < 0.001; DWB, *p* < 0.001), PR (BD, *p* < 0.001; DWB, *p* < 0.001), and PE (BD, *p* < 0.001; DWB, *p* < 0.001) in the BD group and DWB group were significantly higher than those in the UD group. Compared with the HC group, three patient groups had higher TR, PR, and PE.

There was no significant difference in CC between the BD and DWB groups, neither between UD and HC groups. CC in the BD group (*p* < 0.001) and DWB group (*p* = 0.006) was lower than that in the UD group (BD, *p* < 0.001; DWB, *p* = 0.006) and HC group (BD, *p* < 0.001; DWB, *p* < 0.001). CC of the BD group was lower than that in the DWB group (*p* = 0.010) after being adjusted by age, sex, education years, age of onset, number of episodes, course of disorder, duration of current depressive episode, and HAMA and HAMD-17 scores.

RE and nPE in all three patient groups were significantly higher than those in the HC group. RE and nPE in the BD group were significantly higher than those in the DWB group (RE, *p* = 0.012; nPE, *p* < 0.001) and UD group (RE, *p* < 0.001; nPE, *p* < 0.001). RE and nPE in the DWB group were significantly higher than those in the UD group (RE, *p* < 0.001; nPE, *p* = 0.001).

RC in the BD group was significantly lower than that in the DWB group (*p* = 0.030), UD group (*p* < 0.001), and HC group (*p* < 0.001). Differences in RC between the UD group and DWB groups, as well as the UD group and HC group, were not significant. However, in comparison with the HC group, the DWB group had a lower RC (*p* < 0.001).

There was no significant difference in TCFC among the three patient groups. No significant difference in TCFC was detected between the UD group and HC group, while TCFC in the BD group and DWB group was significantly higher than that in the HC group (BD, *p* = 0.007; DWB, *p* = 0.006). In addition, TCFC in the BD group was significantly higher than that in the UD group (*p* = 0.036) after being adjusted by age, sex, education years, age of onset, number of episodes, course of disorder, duration of current depressive episode, and HAMA and HAMD-17 scores.

The score of PCLR in HC group was significantly higher than that in the three patient groups. PCLR in the BD group was lower than that in the DWB group and UD group (*p* < 0.001), and the DWB group scored lower than the UD group (*p* < 0.005) (Table 3).

**Table 3:** Comparison of WCST among patients with bipolar depression, unipolar depression, depression with bipolarity, and healthy controls (mean ± SD).

	**BD (***n*** = 30)**	**UD (***n*** = 30)**	**DWB (***n*** = 30)**	**HC (***n*** = 30)**	***F*** **^1^**	***p*** **^1^**	***post-hoc*^**1**^**	***F*** **^2^**	***p*** **^2^**	***post-hoc*^**2**^**
TR	126.83, 2.15	108.87, 17.73	122.00, 8.03	91.90, 11.93	55.793	<0.001	BD, DWB>UD>HC	22.716	<0.001	BD, DWB>UD
CC	2.43, 2.05	4.53, 1.78	3.30, 1.92	5.60, 0.93	19.487	<0.001	BD, DWB < UD, HC	16.307	<0.001	BD < DWB < UD
RC	56.37, 11.81	69.30, 9.09	64.07, 10.70	76.20, 7.41	21.502	<0.001	BD < DWB, UD, HC; DWB < HC	14.805	<0.001	BD < DWB, UD
RE	70.43, 13.05	39.57, 21.19	56.87, 17.65	15.37, 7.99	68.122	<0.001	BD>DWB>UD>HC	30.363	<0.001	BD>DWB>UD
TCFC	19.77, 3.75	17.30, 12.44	19.60, 6.86	13.17, 3.92	4.906	0.002	BD, DWB>HC	3.373	0.039	BD>UD
PR	30.03, 4.66	16.60, 11.32	23.23, 14.98	4.13, 4.10	37.378	<0.001	BD, DWB>UD>HC	43.069	<0.001	BD>DWB>UD
PE	25.53, 5.35	15.07, 13.11	24.00, 15.76	5.13, 3.47	23.065	<0.001	BD, DWB>UD>HC	16.819	<0.001	BD, DWB>UD
nPE	44.90, 10.33	24.17, 9.60	33.03, 10.16	10.30, 6.01	75.545	<0.001	BD>DWB>UD>HC	10.903	<0.001	BD>DWB>UD
PCLR	20.58, 8.18	56.43, 18.61	45.98, 20.59	72.92, 8.76	63.227	<0.001	BD < DWB < UD < HC	29.743	<0.001	BD < DWB < UD

*BD, bipolar depression; UD, unipolar depression; DWB, depression with bipolarity; HC, healthy control; WCST, Wisconsin Card Sorting Test; TR, total number of response; CC, number of categories completed; RC, total number of response correct; RE, total number of response errors; TCFC, trails to completed first category; PR, perseverative responses; PE, perseverative errors; nPE, non-perseverative errors, PCLR, percent conceptual level responses*.

1 *Age, sex, and education years as covariates among four groups*.

2 *Age, sex, education years, age of onset, number of episodes, course of disorder, duration of current depressive episode, and HAMA and HAMD-17 scores as covariates among three patient groups*.

### Continuous Performance Test

#### Two- and Three-Digit Numbers

There were no significant differences in LR, FR, and MRT among three patient groups, while LR and FR in the three patient groups were higher than those in the HC group. MRT in the three patient groups was longer than that in the HC group, except for the differences of LR in two-digit numbers between BD and HC, which were not significant.

#### Four-Digit Numbers

There were no significant differences in LR, FR, and MRT between the BD and DWB groups; so was FR between the UD and DWB groups. LR in the BD group (*p* = 0.013) and DWB group (*p* = 0.027) was significantly more than that in the UD group. Moreover, the LR and FR of three patient groups were higher than those in the HC group. The difference of MRT among three patient groups was not significant. LR, FR, and MRT in the three patient groups were significantly higher than those in the HC group (BD, *p* < 0.001; UD, *p* = 0.001; DWB, *p* < 0.001) (Table 4).

**Table 4:** Comparison of CPT among patients with bipolar depression, unipolar depression, depression with bipolarity, and healthy controls (mean ± SD).

	**BD (***n*** = 30)**	**UD (***n*** = 30)**	**DWB (***n*** = 30)**	**HC (***n*** = 30)**	***F***	***p***	***post-hoc*** **^1^**	***F*** **^2^**	***p*** **^2^**	***post-hoc*** **^2^**
**2-digit numbers**										
LR	3.33, 3.93	4.10, 3.75	4.80, 4.39	1.40, 1.52	5.061	0.005	DWB>HC	0.429	0.653	
FR	3.00, 2.35	3.00, 3.07	2.33, 1.67	0.67, 1.03	7.740	<0.001	BD, DWB, UD>HC	0.424	0.656	
MRT (ms)	507.67, 53.34	534.37, 66.39	518.23, 62.98	432.52, 38.92	19.207	<0.001	BD, DWB, UD>HC	1.300	0.278	
**3-digit numbers**										
LR	5.60, 3.62	6.27, 4.11	6.33, 4.37	2.40, 1.78	7.869	<0.001	BD, DWB, UD>HC	0.977	0.381	
FR	2.83, 0.38	2.50, 2.86	2.23, 1.76	0.97, 1.16	6.256	0.001	BD, DWB, UD>HC	0.177	0.838	
MRT (ms)	533.67, 51.58	560.70, 64.80	562.99, 92.80	482.73, 58.12	8.865	<0.001	BD, DWB, UD>HC	2.187	0.119	
**4-digit numbers**										
LR	11.77, 4.24	8.57, 3.88	11.53, 4.29	3.53, 3.38	28.022	<0.001	BD, DWB>UD>HC	14.339	<0.001	BD, DWB>UD
FR	7.00, 4.55	4.23, 3.47	6.10, 2.25	1.03, 1.38	21.028	<0.001	BD, DWB>UD>HC	6.430	0.003	BD, DWB>UD
MRT (ms)	617.10, 43.12	608.78, 75.73	627.29, 104.29	528.69, 76.33	10.060	<0.001	BD, DWB, UD>HC	2.288	0.108	

*BD, bipolar depression; UD, unipolar depression; DWB, depression with bipolarity; HC, healthy control; CPT, continuous performance test; LR, leakage responses; FR, false responses; MRT, mean reaction time*.

1 *Age, sex, and education years as covariates among four groups*.

2 *Age, sex, education years, age of onset, number of episodes, course of disorder, duration of current depressive episode, and HAMA and HAMD-17 scores as covariates among three patient groups*.

## Discussion

Our study applied three cognitive test tools to evaluate cognition function among BD, UD, DWB, and HC groups. For THINC-it, the differences of the Z-scores in both objective and subjective parts between DWB and BD were not significant; Z-scores of the BD group were lower than those of the UD group. For WCST, differences in the TR, CC, TCFC, PR, and PE between the BD and DWB groups were not significant. All the indices of WCST in the DWB group were worse than those of the UD group except for TCFC and RC. For CPT, only leakage responses and false responses in the four-digit number of the BD and DWB groups more than the UD group, and the difference between BD and DWB were not significant.

THINC-it is the first tool that provides both objective and subjective cognition tests, and its test domain includes attention, executive function, and memory. To the best of our knowledge, this was the first study to compare cognitive deficits in BD, UD, and DWB by THINC-it. As for each item of objective component, CRT is applied to assess attention and executive function; N-Back evaluates working memory, executive function, and attention/concentration; DSST is used to identify executive functions, processing speed, and attention/concentration; TMT-B tests executive function. The Z-scores of all objective items of three patient groups were lower than those of HC, except for the CRT, and the differences of CRT among the four groups and N-Back among three patient groups were not significant. The Z-scores of DSST and TMT-B in the BD group were lower than those in UD and DWB groups. When integrating four objective items, the differences of Z-scores between DWB and BD, UD and DWB, and UD and HC were not significant, while Z-scores of the BD group were lower than those of the UD group, and Z-scores of BD and DWB were lower than those of HC. As for the subjective component, the difference of Z-score between DWB and BD, and DWB and UD was not significant, while Z-scores of BD were lower than those of UD, and Z-scores of three patient groups were lower than those of HC. The Z-scores of DWB were lower than those of UD. The finding showed that patients with MDE were all with cognitive impairment; BD and DWB had the more critical cognitive impairment than UD.

For WCST, all the indices of WCST in patient groups were worse than those of the HC group except for TCFC (differences of TCFC between UD and HC were not significant). There were no significant differences in TR, CC, PE, and PR between the BD group and DWB group; other indices of WCST in the BD group were worse than those of the DWB group. All the indices of WCST in the DWB group were worse than those of the UD group except for RC and TCFC. It indicated that executive function deficits in patients with MDE were worse than those of HC, deficits of executive function of patients with DWB were more similar with those of patients with BD, and deficits of executive function of patients with BD and DWB were worse than those of patients with UD. Some previous studies showed that the patients with BD in WCST were worse than those with UD and HC, even in the euthymic stage, which was in accordance with our study (25, 26). However, in the study of Lin et al. (22), the differences of indices of WCST between UD and SBP were not statistically significant, as well as between BD and SBP, which was inconsistent with this study. Another recent study also showed only minor differences in executive function between drug-naïve patients with bipolar depression and unipolar depression (27). There might be two explanations for the discrepancy: first, the criteria in the study of Lin et al. were based on the criteria proposed by Akiskal et al., although, the difference of cognition deficit between patients with SBP and patient with DWB was not significant in their study, and their criteria were based on DSM-IV; the heterogeneity of the sample between the two studies was unavoidable. Second, in the study of Lin et al., they just chose three indices of WCST to make a comparison; the differences might be detected when they use more indices.

CPT is applied to assess sustained attention and vigilance (28). In this study, all the indices of CPT in the three patient groups were worse than those in the HC group; there were no significant differences in LR, FR, and MRT of two-digit numbers and three-digit numbers among three patient groups; when it comes to four-digit numbers, the difference of MRT among three patient groups was not significant, differences of LR and FR between the BD group and DWB group were not significant, and LR and FR of the BD and DWB groups more than those of the UD group. The finding suggested that patients with MDE were attention deficit, the difference of extent of attention-deficit among three patient groups could not be detected when the task was easy, and the difference tended to be significant with the difficulty of the task increasing. Previous studies suggested impaired sustained attention present in both the euthymic stage and depressive stage, and it appeared specific to bipolar disorder (29–31).

In our study, some cognition indicators of BD were more severe than those of UD, while differences of other indicators were not significant, which were in accordance with the patterns of brain activity alterations. Recently, a voxel-based meta-analysis showed that UD and BD shared increased amplitude of low-frequency fluctuation (ALFF) in the bilateral insula (a cortical structure with extensive connections to many areas of the cortex and limbic system, which is implicated in disparate cognitive, affective, and regulatory functions) and right medial prefrontal cortex (mPFC; a critical neuronal region in regulating attention, cognitive control, motivation, and emotion), and decreased ALFF in the left cerebellum posterior lobe, suggesting that altered intrinsic activity in these regions is common to both disorders. However, they also find that increasing ALFF of the right insula was significantly greater in BD than MDD, which suggested that the impairment of cognition function in BD might be more severe than that in UD. Moreover, several regions, including the limbic system and occipital cortex, differed between conditions, indicating that these disorders may be associated with spatially distinct patterns of brain function (32). A previous triple-network model study (involving the default mode network, central executive network, and salience network, which is associated with cognitive function, such as attention and working memory) also provided evidence about the shared and specific functional and structural alterations in BD and MDD (33).

The conception of bipolarity is not mentioned frequently because it is a series of clinical characteristics that have not been validated; however, some of them are often associated with bipolar disorder. A guideline proposed by Stahl et al. mentioned that MDE patients with the characteristics, which were coincident with bipolarity, were more likely to convert into bipolar disorder and were at risk of adverse reactions to antidepressant treatments (34). At present, little research about the neurocognitive function of patients with DWB was conducted. The study suggested that patients with DWB were similar to patients with BD in neurocognitive function impairment, which reinforced the concern that patients with UD who manifested bipolarity but did not meet the criteria of bipolar disorder based on the DSM system were actually “bipolar enough” and at risk of inappropriate antidepressant therapy.

As for clinical characteristics, times of mood episode and duration of disorder in BD were more than those in the UD group, so did to times of mood episode between the BD and DWB groups, while the differences between UD and DWB were not significant, which indicated that DWB was too hard to distinguish from bipolar disorder and MDD, especially in depression episode. MDQ, a screening tool for bipolar disorder with established sensitivity and specificity (35, 36), meets the need for distinguishing bipolar patients from patients with MDE. In the present study, there was no significant difference in MDQ score between the DWB and BD groups, and the MDQ score of the BD and DWB groups was higher than that of UD, which reinforced that patients with DWB were similar to patients with BD.

Some limitations of this study should be noted. First, this was a cross-sectional study, and the effect of disorder progression and psychotropic on cognition cannot be explored, as well as the differences of cognition among patients with BD, UD, and DWB in remission state. Second, the sample size of our study was relatively small; larger samples are needed to validate our results in the future. Third, the study of Simonsen et al. (37) found that neurocognition between bipolar I and bipolar II disorder was significantly different, but we did not conduct the subtype stratification analysis result from our small sample size.

In summary, patients with MDE were with cognition impairment; patients with DWB might perform differently from those with UD but similarly to those with BD with cognition impairment. Our finding provides evidence that bipolar disorder may have a distinct neurobiological basis compared with strict UD and may help clinicians better understand DWB patients. Given the limitations of the present study, large-sample longitudinal studies for cognition function in DWB patients are required for future validation.

## Data Availability Statement

The raw data supporting the conclusions of this article will be made available by the authors, without undue reservation.

## Ethics Statement

The studies involving human participants were reviewed and approved by Clinical Research Ethics Committee of Shandong Mental Health Center. The patients/participants provided their written informed consent to participate in this study.

## Author Contributions

ZL and GX designed the study and wrote the paper. ZL and YW analyzed data. All authors participated in the step of enrollment and discussed the results, approved the final version, and can certify that no other individuals not listed as authors have made substantial contributions to the paper.

## Conflict of Interest

The authors declare that the research was conducted in the absence of any commercial or financial relationships that could be construed as a potential conflict of interest.

## Publisher's Note

All claims expressed in this article are solely those of the authors and do not necessarily represent those of their affiliated organizations, or those of the publisher, the editors and the reviewers. Any product that may be evaluated in this article, or claim that may be made by its manufacturer, is not guaranteed or endorsed by the publisher.
